# Costs and consequences of different chemotherapy regimens in metastatic colorectal cancer

**DOI:** 10.1038/sj.bjc.6600273

**Published:** 2002-06-05

**Authors:** J P Hale, D R Cohen, T S Maughan, R J Stephens

**Affiliations:** University of Glamorgan, School of Care Sciences, Pontypridd CF37 1DL, UK; Velindre Hospital, Whitchurch, Cardiff CF4 7XL, UK; MRC Clinical Trials Unit, 222 Euston Road, London NW1 2DA, UK

**Keywords:** costs, chemotherapy, colorectal cancer

## Abstract

An economic sub-study was run alongside a large multi-centre randomised trial (MRC-CR06) comparing three chemotherapy regimens; de Gramont, Lokich and raltitrexed in patients with metastatic colorectal cancer. Patients in six of 45 centres in the main trial were approached to take part in the sub-study. Chemotherapy delivery costs were assessed in each sub-study centre with external validity verified by questionnaire to all other centres. Patient representativeness was assessed. Stochastic resource use data, including patient borne costs and non-hospital health service resource use were monitored prospectively. Mean total societal costs were de Gramont=£5051 (s.d. £1910), raltitrexed=£2616 (s.d. £991) and Lokich=£2576 (s.d. £1711). In pairwise comparisons, statistically significant mean total cost differences were shown for de Gramont *vs* Lokich (mean difference=£2475, 95%CI £914–£4037, *P*<0.01) and for de Gramont *vs* raltitrexed (mean difference=£2435, 95%CI £922–£2948, *P*<0.01). Sensitivity analyses showed little effect on overall costs. The main trial showed de Gramont and Lokich to be equally effective in terms of survival, quality of life and response rates but Lokich had higher toxicity and hand-foot syndrome. Raltitrexed showed similar response rates and overall survival but increased toxicity and inferior quality of life making it a clinically inferior regimen despite its ease of administration and costs. For a comparable clinical outcome, Lokich can be administered for approximately half the cost of de Gramont.

*British Journal of Cancer* (2002) **86**, 1684–1690. doi:10.1038/sj.bjc.6600273 www.bjcancer.com

© 2002 Cancer Research UK

## 

Colorectal cancer kills about 20 000 people each year in the UK. Once the disease has spread beyond the limits of surgical cure, the aim of treatment is to improve quality of life and survival. In a meta analysis of randomised controlled trials, chemotherapy has been shown to improve median survival from 8 to 11.7 months (Colorectal Cancer Collaborative Group, 2000).

A large nation-wide multi-centre randomised trial (MRC-CR06) has been undertaken to compare three chemotherapy treatments for patients with advanced colorectal cancer: de Gramont bolus and infusion 5FU plus folinic acid, Lokich Protracted Venous Infusion 5FU, and raltitrexed (Tomudex™) (Maughan *et al*, 2002). There is, however, a growing awareness that health care resources are scarce and therefore as well as assessing comparative effectiveness in terms of clinical outcomes it is also important to consider at what cost these outcomes are achieved. In addition to the direct costs of chemotherapy delivery, regimens can differ in terms of indirect costs, for example the treatment of serious adverse events or in travel and other costs borne by patients and their carers.

An economic sub-study using a societal perspective was run alongside the clinical trial. A cost effectiveness analysis of the three regimens would have required specification of a single outcome as reflecting the objective of the treatments. Within CR06 a number of outcomes were assessed including overall survival, progression free survival, response rate, treatment related deaths, toxicity, and quality of life using EORTC-QLQ C30, HADS and trial specific questions. As cost effectiveness analyses cannot handle programmes with multiple objectives (Drummond *et al*, 1997) we here report relative costs and consequences of the three regimens over the initial 12 week period.

## MATERIALS AND METHODS

Details of the design of the main trial are available elsewhere (Maughan *et al*, 2002). Briefly, 905 patients with advanced metastatic colorectal carcinoma for whom palliative chemotherapy was the only remaining active treatment option, were randomised to receive de Gramont (dl-folinic acid 100 mg m^−2^, 5-fluorouracil (5FU) bolus 400 mg m^−2^ and infusion 600 mg m^−2^ day 1 and 2, q14d), Lokich (protracted venous infusion of 5FU 300 mg m^−2^ day^−1^) or raltitrexed (3 mg m^−2^ i.v. q21d) for an initial period of 12 weeks.

The economic study was undertaken on a sub-sample of patients from six of the 45 centres taking part in the main trial, selected to represent a geographical spread across the UK. Each centre provided all three chemotherapy regimens. Study patients in the main trial who were receiving treatment in these six centres were invited to participate in the economic sub-study via separate informed consent.

Costs of delivering chemotherapy were estimated via a detailed costing exercise undertaken in each centre in the sub-study. To verify representativeness, a questionnaire on methods of delivering chemotherapy was posted to research nurses and pharmacists in all other centres participating in the main trial. Representativeness of patients in the economics sub-study was assessed by comparing demographic and other characteristics of sub-study patients with all patients in the main trial.

### Costing methods

Costing was undertaken from a societal perspective in which all costs were considered regardless of who bore them. Stochastic resource data (i.e. which were expected to vary by patient) were collected prospectively over the 12 week period following randomisation. A research nurse in each of the six centres monitored patient notes for investigations undertaken and all additional treatments received. Patient borne costs were recorded in diaries by patients, or where necessary by a relative. Weekly entries were made listing all costs incurred by patients and their families, including travel and time taken off work by others to care for the patient. Use of non-hospital health and social care resources (general practitioner, district nurse and other health or social service) were also recorded in the diaries.

Monetary valuation of resource use was undertaken by attaching unit costs to recorded quantity data. All costs are in 1998/99 prices. In the case of all chemotherapy drugs, costs have been calculated on the assumption that any drug remaining in partly used vials will be discarded. Unit costs for drugs are from the British National Formulary (British National Formulary, 1998) which do not reflect any bulk purchase discounts which may be negotiated. Cost of GP and district nurse visits are from Netten *et al* (1998). Costs of in-patient stays, investigations, materials and disposables were provided by the Finance department of one of the participating centres. The opportunity cost of time taken off work by carers was on the basis of average hourly wages (Office for National Statistics, 1999). Robustness of results were assessed via a series of one-way sensitivity analyses. Total societal costs were assessed against outcome data from the whole trial population on survival and quality of life.

### Statistical methods

Independent samples *t*-tests (α=0.05) were used to test for differences in mean costs in pairwise comparisons of the three regimens. Representativeness of the sub-sample was assessed by comparing demographic and other characteristics with all other CR06 patients using χ^2^. Details of the statistical methods used on clinical outcome data are available elsewhere (Maughan *et al*, 2002).

## RESULTS

### Representativeness of sub-sample patients

A sub-sample of 68 patients was obtained. There do not appear to be any differences between this sub-sample and all other patients in the trial as shown in Table 1

**Table 1:**
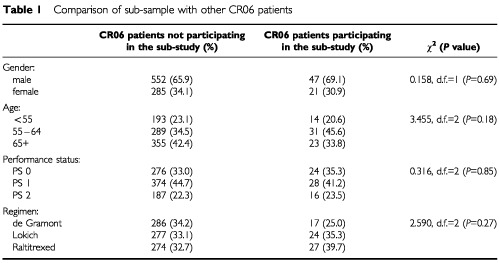
Comparison of sub-sample with other CR06 patients

.

The survival pattern of patients in the economics sub-study was similar to that of those not included (hazard ratio=0.97, 95%CI 0.74–1.27, *P*=0.81). In terms of serious adverse events, patients in the economics sub-study were generally representative of the whole, and although no fatal or life threatening chemotherapy related events occurred, a higher than expected proportion of patients required hospitalisation.

### Representativeness of methods of chemotherapy delivery in sub-study centres

After one reminder, a response rate of 90% was achieved for the pharmacist questionnaire and 95% for the research nurse questionnaire in centres which did not participate in the sub-study.

Table 2

**Table 2:**
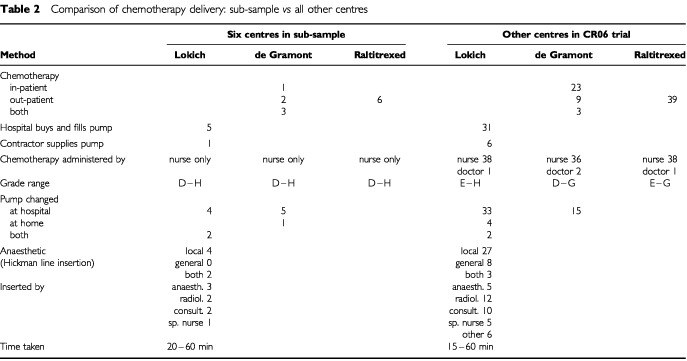
Comparison of chemotherapy delivery: sub-sample *vs* all other centres

compares sub-study with other centres. This was a pragmatic trial and during the study period some centres began to provide de Gramont to some patients on an out-patient basis. The proportion of sub-study centres which provided de Gramont on an out patient basis was broadly similar to the other centres (two of six in the sub-study *vs* nine of 35 in the other centres who reported). Table 2 also shows sub-study centres to be broadly representative in terms of a number of other variables.

### Mean total societal costs

Mean total societal costs were highest for de Gramont (£5051, s.d.=1910), followed by raltitrexed (£2616, s.d.=991) and Lokich (£2576, s.d.=1711) as shown in Table 3

**Table 3:**
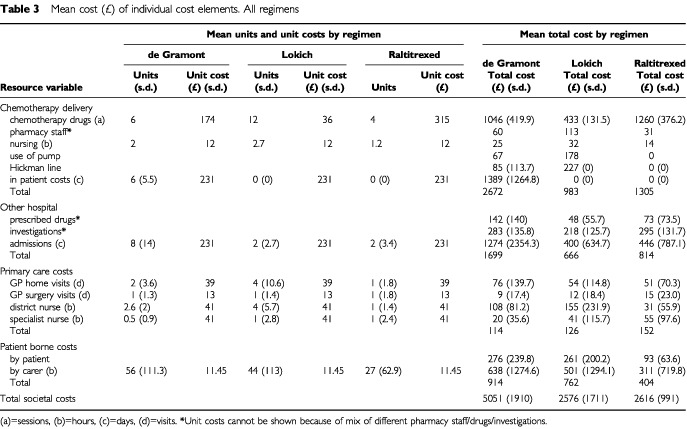
Mean cost (£) of individual cost elements. All regimens

. Costs were based on the assumption that all patients in the Lokich arm, and all who received de Gramont as out-patients, used Hickman, rather than other central venous access devices.

With regard to de Gramont, the costs shown reflect the way that chemotherapy was actually delivered within the trial i.e. with a mix of in-patient (63%) and out-patient (37%) treatment. Cost of chemotherapy drugs (£1046) represented 39% of the cost of de Gramont delivery (£2672) which in turn made up 53% of total societal costs (£5051). In-patient admissions other than for chemotherapy (£1274) represented a further 25% of total societal costs with patient borne costs (£914) representing 18%.

In the case of Lokich, cost of chemotherapy drugs (£433) represented 44% of the cost of chemotherapy delivery (£983) which in turn represented 38% of total societal costs (£2576). In-patient admissions (£400) were responsible for a further 16% of total societal costs with patient borne costs (£762) representing 30%.

By contrast, the cost of raltitrexed drugs (£1260) represented almost the whole (97%) of chemotherapy delivery (£1305) which in turn made up 50% of total societal costs (£2616). In-patient admissions (£446) represented a further 17% with patient borne costs (£404) lower than the other regimens and representing 15%.

### Differences in total costs and individual cost elements

Total societal costs are made up of 15 individual cost elements. Mean differences and confidence intervals for those variables where these differences were statistically significant (pairwise comparisons) are given in Table 4a

**Table 4a:**
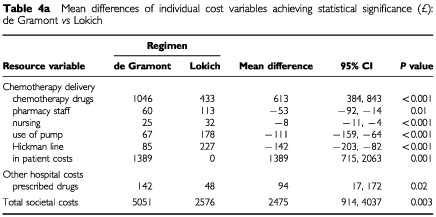
Mean difference of individual cost variables achieving statistical significance (£): de Gramont *vs* Lokich

(de Gramont *vs* Lokich) Table 4b

**Table 4b:**
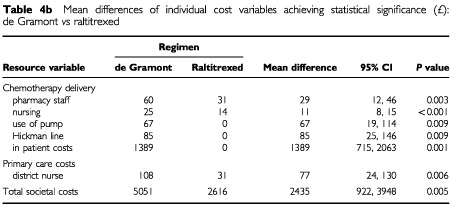
Mean differences of individual cost variables achieving statistical significance (£): de Gramont *vs* raltitrexed

(de Gramont *vs* raltitrexed) and Table 4c

**Table 4c:**
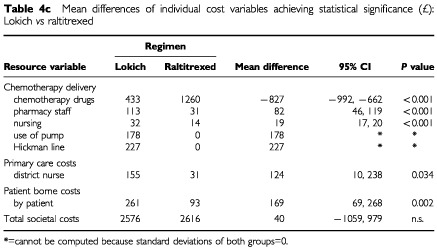
Mean differences of individual cost variables achieving statistical significance (£): Lokich *vs* raltitrexed

(Lokich *vs* raltitrexed). Differences in mean total societal costs between de Gramont and Lokich (£2475) were statistically significant (*P*<0.01) as were those between de Gramont and raltitrexed (£2435, *P*<0.01). Total costs of Lokich and raltitrexed were similar with a mean difference of only £40.

Examination of differences in the mean estimates for each element specifies where the differences in total costs occur. Although there were no significant differences in total societal costs between Lokich and raltitrexed, pairwise comparisons showed statistically significant differences in the cost of chemotherapy drugs (raltitrexed higher, *P*<0.001), pharmacy staff (Lokich higher, *P*<0.001), hospital nurses (Lokich higher, *P*<0.001), district nurses (Lokich higher, *P*<0.05) and patient borne costs excluding carers (Lokich higher *P*<0.01).

### Outcomes

Full clinical results are available elsewhere (Maughan *et al*, 2002) and are summarised in Table 5

**Table 5:**
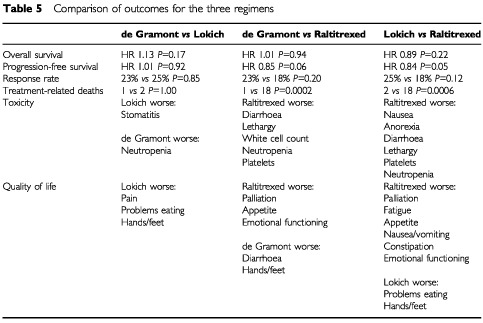
Comparison of outcomes for the three regimens

. The three regimens were broadly equivalent in terms of overall survival and response but there were higher treatment-related deaths in the raltitrexed arm (18 out of 301 *vs* 3 out of 604).

De Gramont and Lokich were broadly equivalent in terms of quality of life, although Lokich patients reported more soreness of the hands and feet. Raltitrexed was inferior to de Gramont in terms of palliation (lack of appetite), role functioning, social functioning and global quality of life. Raltitrexed was also inferior to Lokich in terms of palliation, fatigue, appetite, nausea/vomiting, constipation and emotional functioning.

### Sensitivity analyses

A number of factors were identified that could have implications for the cost of the different chemotherapy regimens. As Table 2 indicates, nurses within the grade range D to H could be responsible for the administration of chemotherapy. The base case analysis assumed the mid point of this range and therefore used the cost of an F grade nurse. It is important, however, to consider the potential impact of either extreme grade of nurse being used to carry out this activity.

Another important difference is the two possible methods of delivery of the de Gramont regimen. The base case analysis calculated the cost of the de Gramont regimen based on a mixture of in-patient and out-patient delivery. There are clearly cost implications if this regimen is always provided on an in-patient or always provided on an out-patient basis.

By the end of the study period, many centres had changed from using Hickman lines in the delivery of the Lokich regimen, to cheaper peripherally implanted central catheters. The impact of this change needs also to be considered.

In the base case analysis, the costs of in-patient stays, investigations, materials and disposables were provided by the Finance Department of one of the participating centres. To take account of possible variations in these figures, the analysis was repeated twice more using extreme values; once assuming that costs were half those in the base case analysis and once assuming they were double.

One final factor worth considering is the impact on nursing time of patients receiving the Lokich regimen being trained to change the pump themselves rather than attending hospital for a nurse to change it.

Tables 6a

**Table 6a:**
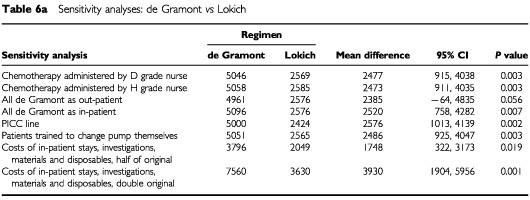
Sensitivity analyses: De Gramont *vs* Lokich

, 6b

**Table 6b:**
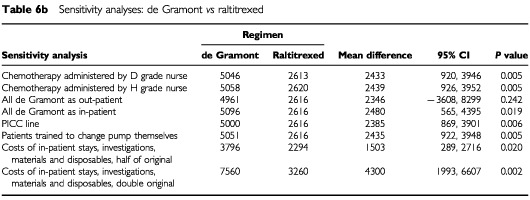
Sensitivity analyses: De Gramont *vs* raltitrexed

, 6c

**Table 6c:**
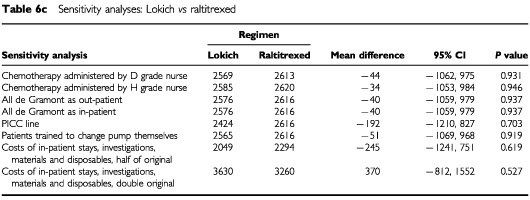
Sensitivity analyses: Lokich *vs* raltitrexed

show the impact of the above changes on the mean total societal costs for each of the pairwise comparisons. The overall results are not altered by any of these changes, with the exception of providing de Gramont solely on an out-patient basis. The differences in total societal costs of providing de Gramont as an out-patient and as an in-patient, may not appear as great as may have been expected. This is likely to be due to the small numbers being considered here. Altering the size of the cost estimates, whilst not effecting the overall results, did have a different impact on the three regimens. When using double cost estimates, the total societal cost of Lokich is higher than that of raltitrexed, although this difference is not statistically significant.

## DISCUSSION

### Costs

This study has demonstrated that over a 12 week period, study patients who received the de Gramont regimen had significantly higher costs in terms of both direct chemotherapy delivery and total societal costs than those receiving either Lokich or raltitrexed.

New drugs usually carry a high drug cost but this study has shown that despite the relatively high cost of raltitrexed, overall societal costs compare favourably with those of the other regimens. This is consistent with Ross *et al* (1996) who also found the total cost of raltitrexed to be comparable to that of continuously administered 5FU and lower than that of de Gramont. Moreover, the lower frequency of attendance associated with the raltitrexed 3 weekly regimen also results in lower patient borne costs. The frequency of chemotherapy delivery also affects the mean costs of both pharmacy and nursing staff. This is in line with the results from other studies which found pharmacy staff costs to be lower for raltitrexed than for other 5FU regimens (Summerhayes *et al*, 1997).

From the hospital perspective the Lokich regimen is the least costly. Although raltitrexed had the lowest costs in terms of pharmacy and nursing time, these were not sufficient to offset the higher drug costs. This study thus provides no evidence to support the results of Kerr and O'Connor (1997) which showed that raltitrexed reduces chemotherapy costs when compared with the Lokich regimen.

As regards the cost of treating chemotherapy related side effects, no significant differences were found in terms of prescribed drugs. This is in contrast to the finding of Elliott (1996) where drug costs for management of side effects were lower for raltitrexed patients as compared with those receiving 5FU plus folinic acid. While the present study showed a trend toward higher in-patient costs for de Gramont (other than to receive chemotherapy) these differences did not reach statistical significance (de Gramont *vs* Lokich, mean difference=£874, *P*=0.181, de Gramont *vs* raltitrexed, mean difference=£828, *P*=0.206), although this must be considered in the light of the small sample size. General practitioner visits, either at home or in the surgery, were also similar suggesting that patients on one regimen are not seeking more help in primary care than patients on any other regimen. Overall, this study provided no evidence that the cost implications of side effects and serious adverse events (requiring prescribed medication or hospital admission) differ between regimens.

### Potential for cost improvement

Clearly, these reported costs can vary by changing the way in which chemotherapy is delivered. For example, providing de Gramont on an out-patient basis avoids in-patient costs and can thus significantly reduce hospital costs. Within this study patients who received de Gramont as out-patients (*n*=6) had chemotherapy delivery costs which were roughly 50% lower (*P*=0.011) than for in-patient delivery. The relatively low degree of statistical significance is likely to be due to the small numbers involved. Chemotherapy delivery, however, only represents a part of total societal costs and de Gramont patients receiving out-patient delivery had higher primary care costs than those receiving it as in-patient (£283 *vs* £85, *P*=0.04). Even on the basis of these very small numbers, use of de Gramont as an out-patient regimen is likely to have important implications for hospital costs.

There is also the possibility of further reducing de Gramont drug costs by modifying the dose. In an ongoing MRC trial (CR08) the de Gramont regimen has been modified to 200 mg m^−2^ folinic acid plus 400 mg m^−2^ 5FU bolus on day 1 followed by 2800 mg m^−2^ 5FU infusion over 46 h. Calculating the cost of the chemotherapy drugs that de Gramont patients in the present sub-study would have received if they had been given this modified regimen shows a significant reduction in mean drug costs from £1046 to £667 during the 12 weeks (*P*<0.001). Delivering modified de Gramont on an out-patient basis will have further implications for hospital costs. Patients who received de Gramont as an out-patient in the present trial still incurred mean in-patient costs for chemotherapy delivery, although these were much lower (£231 *vs* £2084) than for those who received this regimen as an in-patient.

## CONCLUSION

The toxicity and impaired quality of life observed with raltitrexed make it a clinically inferior regimen despite its ease of administration and costs. In making a choice between de Gramont and Lokich, a number of issues need to be taken into consideration given that their overall clinical benefit is equivalent. Lokich is inferior to de Gramont only in terms of the increased incidence of minor adverse events and central line complications. There was no evidence that the management of these events had different cost implications between the two regimens. However, hand and feet soreness, while neither affecting measurable quality of life nor resulting in costs, can be an added burden, if a manageable one. Patients have recorded no difference in terms of interference with daily living between de Gramont and Lokich. However, in current practice modified de Gramont is now administered as an out-patient regimen in nearly all centres. This has the positive effect of reducing interference with normal life and cutting hospital costs, but does mean that all patients now require central lines with the associated incidence of line complications.

In conclusion, in comparison with de Gramont as administered in this study, the significant reduction in costs of Lokich with comparable clinical benefit suggest that it offers the best value for money of the three regimens.
